# Variant site strain typer (VaST): efficient strain typing using a minimal number of variant genomic sites

**DOI:** 10.1186/s12859-018-2225-z

**Published:** 2018-06-11

**Authors:** Tara N. Furstenau, Jill H. Cocking, Jason W. Sahl, Viacheslav Y. Fofanov

**Affiliations:** 1 The School of Informatics, Computing, and Cyber Systems, Northern Arizona University, 1295 S Knoles Dr., Flagstaff, Arizona, 86001 USA; 2 Pathogen and Microbiome Institute, Northern Arizona University, 1395 S Knoles Dr., Flagstaff, Arizona, 86001 USA

**Keywords:** Targeted PCR Amplicon sequencing, Bacterial strain typing, Single nucleotide polymorphisms

## Abstract

**Background:**

Targeted PCR amplicon sequencing (TAS) techniques provide a sensitive, scalable, and cost-effective way to query and identify closely related bacterial species and strains. Typically, this is accomplished by targeting housekeeping genes that provide resolution down to the family, genera, and sometimes species level. Unfortunately, this level of resolution is not sufficient in many applications where strain-level identification of bacteria is required (biodefense, forensics, clinical diagnostics, and outbreak investigations). Adding more genomic targets will increase the resolution, but the challenge is identifying the appropriate targets. VaST was developed to address this challenge by finding the minimum number of targets that, in combination, achieve maximum strain-level resolution for any strain complex. The final combination of target regions identified by the algorithm produce a unique haplotype for each strain which can be used as a fingerprint for identifying unknown samples in a TAS assay. VaST ensures that the targets have conserved primer regions so that the targets can be amplified in all of the known strains and it also favors the inclusion of targets with basal variants which makes the set more robust when identifying previously unseen strains.

**Results:**

We analyzed VaST’s performance using a number of different pathogenic species that are relevant to human disease outbreaks and biodefense. The number of targets required to achieve full resolution ranged from 20 to 88% fewer sites than what would be required in the worst case and most of the resolution is achieved within the first 20 targets. We computationally and experimentally validated one of the VaST panels and found that the targets led to accurate phylogenetic placement of strains, even when the strains were not a part of the original panel design.

**Conclusions:**

VaST is an open source software that, when provided a set of variant sites, can find the minimum number of sites that will provide maximum resolution of a strain complex, and it has many different run-time options that can accommodate a wide range of applications. VaST can be an effective tool in the design of strain identification panels that, when combined with TAS technologies, offer an efficient and inexpensive strain typing protocol.

## Background

High-resolution strain identification is vital in applications ranging from tracking of disease outbreaks and surveillance of virulent or antimicrobial resistant pathogens [1–3] to the investigation of bioterrorism and other crimes [4–6]. One of the most promising methods for molecular-based strain identification is targeted multiplex PCR amplicon sequencing (TAS) using high throughput sequencing (HTS) platforms [7]. From an unknown isolate, targets are amplified together in a multiplexed PCR reaction and sequenced, the sequences are then analyzed and compared to sequences of known isolates for identification. PCR enrichment of target sequences allows TAS to be more cost effective than whole genome sequencing and tolerant to low amounts of starting material [8]. Combining this with HTS technology allows scaled processing of hundreds to thousands of samples on a single machine. The challenge is then deciding which targets to choose to achieve the desired outcome.

The targeted sequences have often been either a single housekeeping gene (e.g. the 16S rRNA gene [9]) or in the case of multi-locus sequence typing (MLST), a collection of a few housekeeping or well-conserved genes [10]. The variation within these genes is used to define a well curated set of different sequence types (ST) that distinguish bacterial species or strains. Depending on the amount of diversity, MLST can provide decent resolution and, as HTS techniques are increasingly applied, it is becoming more scaleable and cost-effective [11]. For some applications, however, the resolution from only a few genes can be insufficient, especially for differentiating between closely related or highly clonal variants [12]. When identifying genetic variation that distinguishes specific strains there is not always enough variation found among the established targets.

VaST was designed to find a minimal set of target loci that provide a desired level of resolution across a given strain complex. It can add resolution to an existing MLST assay or it can generate a complete set of targets from scratch when MLST loci have not been established. Either way, the goal of VaST is to provide flexibility and control to the design of specialized strain-typing assays for a number of different applications that can be customized for specific sequencing technologies. This begins with the user defining the level of strain resolution that they desire from the panel. If resolution among a specific group of strains is particularly important, this can be defined and VaST will focus on maximizing resolution for those strains. Next, established targets of variation (such as loci from a MLST assay [10, 13–19] or canonical SNPs [20–33]) can be added as a starting point which will override the VaST optimization function to guarantee their inclusion in the final set. Other targets, such as those associated with virulence or antimicrobial resistance can also be included. VaST will search for additional targets, considering many different types of genetic variation including: single nucleotide polymorphisms (SNPs), microsatellites, variable number tandem repeat (VNTRs), and small insertion/deletions (indels). These targets will be contained within a user-specified amplicon size that is appropriate for the desired sequencing technology. Because the selected targets must be amplifiable across all the strain variants, VaST will pre-filter any target that does not have sufficiently well conserved flanking primer sequences. VaST will identify and add new targets until either maximum resolution is reached, a predetermined resolution level is reached, or a specified number of targets have been identified.

Finding the minimal number of targets to achieve the desired resolution is important because it keeps costs low and it limits the potential for adverse primer interactions during multiplex PCR. Given a set of variable genomic sites to choose from, this task is, in essence, a minimum spanning set problem — the minimum set of genomic features that is capable of uniquely identifying each strain. Naively, one would hope to find a single polymorphic site per strain that uniquely distinguishes it from all other strains. In practice, finding a signature polymorphism for each strain is unlikely and the significance of such a signature may erode when additional strains are considered. Instead, our approach seeks to identify a “haplotype” or a collection of polymorphisms which in concert, provide a composite signature that is unique for any given strain. The resulting set of targets needs to be robust enough to proactively handle the rapid expansion of sequences for new strains that come with the genomic age. For this reason, we believe that the best set of targets should include basal genomic features that are stable across entire clades of strains and allow accurate placement of strains that have not been seen before. Our minimum spanning set algorithm selects each new target site based on its ability to evenly split up groups of unresolved strains. An important aspect of evenly splitting the strain complex at each step is that the early additions to the minimum spanning set tend to be more phylogenetically basal. Due to an abundance of “deep” phylogenetic markers, our approach, as we demonstrate, is very robust to characterizing previously unseen strains.

Several groups have developed approaches for identifying a minimum set of target markers for various purposes. Pan-PCR [34] and the Loci Selector Module of PanSeq [35] are the most *thematically* similar approaches as they both focus on strain typing; however, there are other methods which focus on different problems like finding a minimum set of haplotype tagging Single Nucleotide Polymorphisms (htSNPs) for identifying haplotype blocks [36–40]. The Pan-PCR algorithm uses whole genome sequence data from closely related strains to find a minimum number of gene targets whose presence or absence in a PCR product can be used to distinguish a set of input strains. Primers are designed specifically for each target to ensure that they produce different sized PCR products and the amplified targets are separated in a gel, producing a unique banding pattern that acts as a fingerprint for each of the strains of interest. In contrast, VaST’s minimum spanning set algorithm is able to take advantage of variation that exist in both coding and non-coding regions of the genome which provides a larger pool of options for strain differentiation. This is critical when expanding this approach to viral organisms. VaST is also intended to be used in a sequencing-based approach which will maximize the information content of polymorphic sites, making it possible to detect presence of previously unseen strains and to place them within existing phylogenies. The Loci Selector (LS) module of the PanSeq program is another algorithm which attempts to find loci that offer maximum discriminatory power between certain strains. Like, VaST, the LS module is agnostic with respect to the type of sequence variation that is provided as input. Unlike VaST however, the goal of the LS module is not to find a minimum set of sites that together provide maximum resolution, but rather to find a set (of a provided size) of the most discriminatory loci that have the least amount of overlap. In this case, loci that are “deeper” in the phylogeny are not prioritized because they resolve clades rather than individual strains. The resulting set of targets provides strain resolution but are less robust to correctly placing “new” strains – those not part of the original panel.

In this paper we present the VaST algorithm which computes a minimum set of targets for the purpose of bacterial strain differentiation. We provide benchmarks, computational and experimental validation, and resolution comparisons to the LS module of PanSeq and MLST assays to demonstrate how VaST can help streamline the development of fast, efficient, and cost-effective strain identification assays.

## Implementation

VaST is written in Python and is designed to convert a set of genomic features from different strains into a minimum spanning set of targets which will achieve a maximum (or user-defined) level of strain differentiation. The set of genomic features can be identified using a number of available software packages that detect variant sites across a collection of genomes (we utilized NASP, a single nucleotide polymorphism (SNP) detection pipeline [41]). VaST accepts a variant site matrix where each row represents a genomic site that varies across the columns of strains; the values in the matrix characterize the state of each strain at the variable sites (See example in Table 1). Many different types of genomic variation can be included in this matrix (SNPs, indels, VNTRs, etc.) provided that the variable region is short enough to be captured in a single amplicon.

**Table 1:** SNP Matrix example

LocusID	Strain A	Strain B	Strain C	Strain D	Strain E
genome123::115::115	A	T	A	A	T
genome123::120::120	G	C	G	G	C
genome123::121::121	T	C	C	C	C
genome123::130::130	C	G	G	G	C
genome123::209::209	A	C	C	N	G
genome123::405::405	-	-	X	C	C
genome123::511::541	10	8	8	10	8
⋮	⋮	⋮	⋮	⋮	⋮

The first column of a variant site matrix contains a genome identifier, a start position, and an end position, each separated by two colons. The start and end position should be the same for SNPs. Each additional column represents a strain and the calls made at each variant site for that strain. The first five rows contain SNPs, the sixth row contains an indel with missing data for Strain C, and the last row contains the lengths of VNTRs (the stopping position is based on the longest repeat of 3 in this case)

VaST is able to correctly interpret variant site matrices that contain missing data and ambiguous base calls; although, such sites can slow down the processing of the matrix. To speed up the preprocessing, VaST can be run in a strict mode which will ignore any site with ambiguous or missing data. By default, missing data is represented by an “X”, and deletions are represented by a “-”, and VNTRs can be represented by the number of repeats. The only other permissible character states in the matrix are DNA bases and IUPAC ambiguous base codes [42].

**Fig. 1:**
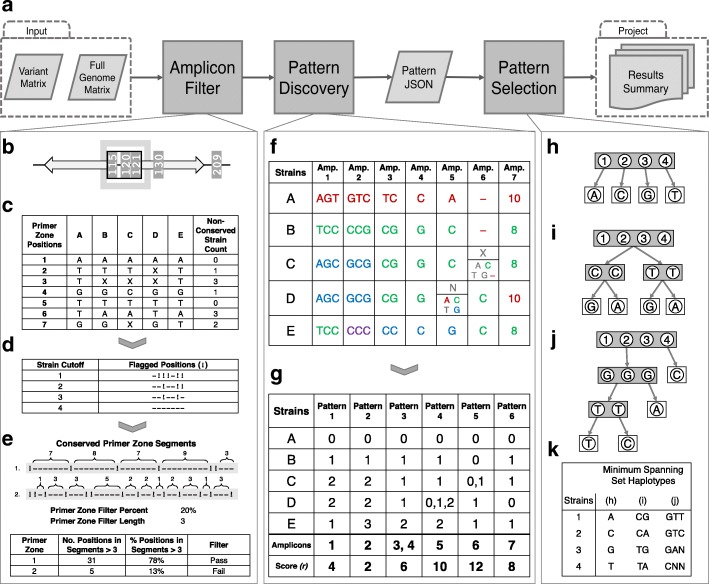
VaST Pipeline Schematic. **a** Overview of the VaST pipeline. **b** The window (gray box) starts at the first site (115) and captures two additional sites (120 and 121). The amplicon (black box) extends from the first to the last variant site in the window and the primer zones (arrows) extend in opposite directions. **c** The primer zone region is extracted from the full genome matrix and the number of strains that are missing data (X) or have a base call that differs from the reference are counted for each position. **d** A position in the primer zone is flagged (!) when the number of poorly conserved strains is greater than or equal to the strain cutoff value. **e** To pass the filter in this example, 20% of the primer zone positions must be a member of a conserved segment that is longer than three positions. **f** The table shows the variant sequence features of the amplicons **g** The resolution pattern of each amplicon is determined and the amplicons that contain redundant information are combined (e.g. Amplicon 3 & 4 into Pattern 3). For ambiguous (N) or missing calls (X), all of the possibilities are enumerated and the strain simultaneously belongs to all of the feature categories that overlap with those of the other strains. The bottom row is the resolution score, *r*, for each pattern. The minimum spanning set algorithm favors patterns that evenly split up groups of strains. Using SNPs as an example, **h** is the best case scenario where *N* strains can be resolved with log4(*N*) SNPs; however, **i** log2(*N*) is more likely with bi-allelic SNPs. **j** In the worst case, highly unbalanced splitting can occur which can require at most *N*−1 SNPs to resolve *N* strains. **k** The associated haplotypes for each of the minimum spanning sets in (**h**-**j**)

To run the Amplicon Filter Module (Fig. 1a), VaST requires information about the regions upstream and downstream of each of the variant sites. Therefore, a full genome matrix must be provided which should include a call for each position in the genome for all of the strains. This matrix can be generated through the alignment of genome assemblies to a reference genome or from Variant Call Format (VCF) files [43] that contain calls for each position in the genome.

### Finding candidate amplicons from target sites

It is assumed that the target sites identified by VaST will ultimately be amplified using PCR and sequenced. Therefore, we included an Amplicon Filter Module which treats each variant site as a potential amplicon, combining adjacent sites as necessary, and filters out any amplicons that may be difficult to amplify in all strains.

When multiple variant sites are clustered together, it is more efficient to consider them together as a single amplicon which can be amplified with one pair of primers. The combination of sites in such an amplicon may sometimes provide more strain resolution than any one of the sites individually, and these more efficient amplicons will naturally be favored during the VaST Pattern Selection Module (Fig. 1a). The maximum distance between adjacent variant sites is defined by a window size parameter. The window starts at the position of the first variant site, and the algorithm checks to see if any of the next variant sites are captured within the window. If the window contains only the original site, this single target amplicon will be sent to the filtering step. If the window contains multiple variant sites, as shown in Fig. 1b, then the amplicon containing all of the sites will be sent to the filter. If this multi-target amplicon fails the filter, the last target site in the window will be removed and this modified amplicon will be sent to the filter. This will be repeated until either an amplicon passes the filter or there are no more target sites in the amplicon. Once the options at the first position are exhausted, the window shifts down to the next variant site. It is possible for the same region to be captured in multiple amplicons so VaST will avoid choosing overlapping amplicons in the final solution. Customizing window lengths allows VaST to be optimized for a wide range of sequencing platforms, which vary widely in the lengths of genomic sequences that can be produced.

To amplify the target sites in a PCR, primers must be designed to anneal in the regions upstream and downstream of the target. If a single set of primers is to be designed that will amplify the target across all of the strains, the primer region must be well conserved. While VaST does not attempt to design the primers themselves, it does consider the conservation of the upstream and downstream primer regions and filters out targets that contain too much variation. During the filtering step, the proposed upstream and downstream PCR primer zones are analyzed and if they contain too much variation between the known strains (based on the number of strains with an alternative allele), or if there are too many strains with missing data, the amplicon is removed from consideration. This ensures that any remaining target sites will have highly conserved primer zones, and thus, have many options for primer design. The cutoffs for acceptable amounts of variation and number of missing strains are user-defined.

More specifically, amplicon filtering is determined by a number of user-provided parameters: the size of the primer zone, a strain cutoff, a primer zone filter percent, and a primer zone filter length. For each amplicon, the base calls for the upstream and downstream primer zone are retrieved from the full genome matrix (Fig. 1c). For each position in the primer zone, the number of strains with a variation or with missing data are counted and, if the count is greater than or equal to the strain cutoff, the position is flagged (Fig. 1d). The segments of the primer zone that are not interrupted by flagged positions are highly conserved and are appropriate for primer design (Fig. 1e). However, in order to pass the filter, a certain percent (primer zone filter percent) of the primer zone positions must be present in segments that are longer than the primer zone filter length. This ensures that the conserved sections of the primer zone are long and contiguous. The primer zone filter is applied separately to the upstream and downstream primer zones, and both zones must pass the filter in order for the amplicon to remain. Table 2 provides a summary of the parameters required for the Amplicon Filter Module.

**Table 2:** Amplicon Filter Module parameter descriptions and considerations

Parameter	Description	Notes
Strict mode	VaST ignores missing or ambiguous data in input matrix	Speeds up preprocessing but some sites are lost
Window size	Maximum distance between adjacent sites that can be combined into a single amplicon	The desired amplicon length should be considered when setting the window size. A larger window may increase the number of variant sites that are included in the amplicons making them more efficient
Primer zone size	Size of the region upstream and downstream of the target to evaluate in the amplicon filter	The primer zones begin immediately before the first and immediately after the last target site in the window, so the maximum amplicon size is 2 × primer zone size + window size. A smaller primer zone may limit the number of primer options.
Strain Cutoff	The number of strains at a primer zone site that can have a non-conserved call before the site is flagged.	A strain cutoff greater than one will not guarantee that the primer zone sequences are conserved across all of the strains but it may be appropriate in cases where one or a few strains have low sequence coverage
Primer zone filter percent	The percent of primer zone positions that must be present in un-flagged segments of the primer zone that are longer than the primer zone filter length.	A higher primer zone filter percent will increase the total number of primer options in amplicons that pass the filter
Primer zone filter length	The length of un-flagged primer zone segments that count toward the primer zone filter percent	The primer zone filter length should be at least as long as the minimum acceptable primer length to ensure that conserved primers can be found within the primer zone

### Characterizing the discriminatory power of candidate amplicons

A resolution pattern is calculated for each amplicon after it passes the amplicon filter. The resolution pattern describes which strains share the same features for a given amplicon (Fig. 1f). The Pattern Discovery Module maps the vector of strain features, **q**, for each amplicon to a pattern vector, **p**, which contains sets denoting the membership of each strain in a unique feature category (Eq. 1 and Fig. 1g). Strains will typically belong to a single feature category but they may belong to multiple categories when they have ambiguous or missing base calls at the target sites within the amplicon (Fig. 1g, Pattern 4, Strain D). When operating under strict mode, the algorithm can assume that there are no missing or ambiguous calls and Eq. 1 simplifies to Eq. 2.

1 $$ {\selectfont{\begin{aligned} \mathbf{q} &= \left[s_{1}, s_{2}, \dotsc, s_{n}\right];\!\!\!\!\quad \text{where \textit{s} is the set of feature states}\\ &\qquad\qquad\qquad\qquad\quad\!\!\! \text{for each of the \textit{n} strains}\\ \mathbf{p} &= \left[f(s_{1}), f(s_{2}), \dotsc, f(s_{n})\right] \\ {}f\left(s; a = \{\mathbf{q}: \vert s_{i} \vert = 1\}\right) &= \left\{\begin{array}{ll} g(s;a) & \quad \text{if}\,g(s;a) \neq \emptyset\\ f(s;a \cup s) & \quad \text{otherwise}  \\ \end{array}\right. \\ g\left(s; a\right) &= \left\{i \colon a_{i} \cap s \neq \emptyset\right\} \\ \end{aligned}}}  $$

Assuming there are no missing or ambiguous calls, Eq. 1 simplifies to:

2 $$ f_{s}\left(s; a = \{\mathbf{q}\}\right) \mapsto \{i \colon a_{i} \in s \}   $$

Despite differences in the specific sequence information of each amplicon, many amplicons will contain redundant strain differentiating information (e.g. Fig. 1f, Amplicon 3 & Amplicon 4). Therefore, instead of storing all of the amplicons individually, they are grouped together based on their strain resolution pattern (Fig. 1g, Pattern 3). Each of these patterns along with the start and stop positions of their associated amplicons are saved in a JSON file that can be passed repeatedly to the Pattern Selection Module without rerunning the preprocessing steps.

#### Constructing the minimal set of targets

The primary goal of the Pattern Selection Module is to find a minimum spanning set, which we define as the minimum number of patterns that are required to achieve maximum strain resolution. A naive brute-force approach to solving for the minimum spanning set requires an exhaustive search of all possible subsets of variant sites, starting from size 1 to *N* where *N* is the size of the minimum spanning set. In the worst case, this approach has exponential complexity ($\mathcal {O}(2^{n})$), which quickly becomes an intractable problem even for relatively small sets of variant sites. For example, given a set *V* of 1,000 variant sites, the size of the search space, |*S*|, that is required to find a minimum spanning set of size 50 is on the order of 10^85^ combinations — more than the estimated number of atoms in the universe. For reference, a typical SNP matrix for a well-studied bacterial strain complex contains 10-30 thousand SNPs.

3 $$ {\selectfont{\begin{aligned} {}\vert S \vert \,=\, \sum\limits^{N}_{k=1} \frac{\vert V \vert !}{k!\left(\vert V \vert \,-\, k\right)!}; \quad \!\!\! & \text{where}\ V\ \text{is the set of variant sites and}\ N\ \text{is the}\\ &\text{size of the first minimum spanning set.} \end{aligned}}}  $$

Because a brute-force approach is intractable, we take a greedy approach which does not guarantee that the absolute minimum spanning set will be found but it will find a locally-optimal, minimized spanning set in a reasonable amount of time. The minimum spanning set algorithm implemented in VaST takes advantage of the exponential increase in discriminatory power with each additional pattern that is added to the set. For example, a single SNP can differentiate at most three strains because there are 4 DNA bases and at least one of the variants must be repeated for any group of more than four strains. When two SNPs are combined into a haplotype the number of possible combinations increases to 16, and a maximum of 15 strains may be uniquely identified. The discriminatory power increases exponentially at 4^*n*^−1 where *n* is the number of SNPs in the haplotype. In contrast, binary variant (presence/absence or wild-type/mutant) approaches (c.p. [34]) can achieve a maximum discriminatory power of only 2^*n*^−1.

For SNPs, the theoretical minimum spanning set requires log4(*N*) SNPs to resolve *N* strains (Fig. 1h). To achieve this minimum, each SNP must contain all four allelic variants and the variants must evenly split up each group of unresolved strains. In practice, many SNPs are only bi- or tri-allelic so a more realistic minimum would be log2(*N*) which may still be difficult to achieve when working with a limited set of available patterns (Fig. 1i). In the worst case, each SNP is only able to differentiate a single strain which causes highly uneven splitting and can require up to *N*−1 SNPs (Fig. 1j).

In order to get as close as possible to the minimum number of variant sites, VaST favors the addition of sites that do the best job of evenly splitting up the most remaining groups of unresolved strains. In practice, this predisposes VaST to prefer at least some phylogenetically basal variants in its solutions (stable variants that occurred sufficiently far in the organism’s past to be established in multiple clades’ lineages). This confers significant advantages when encountering previously unobserved strains. More specifically, the algorithm iteratively incorporates patterns into the set by choosing the pattern that provides the greatest reduction in the set resolution score, *r*, (Eq. 4, Fig. 1g, bottom row). Before any sites are added, each value in the minimum spanning set pattern vector is zero because all of the strains are members of the same null haplotype category. The resolution score is also set to the maximum value of *N*(*N*−1) where *N* is the number of strains. At the beginning, a resolution score is also calculated for each of the amplicon pattern vectors and they are sorted from lowest (best) to highest (worst). Due to the nature of greedy algorithms, it is likely that pattern choices that are locked in the early stages can lead to a sub-optimal solution. Therefore, a number of the top patterns from the sorted list can be selected to seed several distinct, independently-built sets and the best solution will be returned at the end.

When the first pattern is added, the minimum spanning set pattern vector is updated (Eqs. 5 or 6 in strict mode), the resolution score is recalculated and the selected pattern is removed from further consideration. The remaining pattern vectors are then updated so they reflect their resolution combined with the resolution of the current minimum spanning set (Eqs. 5 or 6) and their scores are recalculated (Eq. 4). The pattern with the best score is then added to the minimum spanning set. Patterns are continually added in this manner until (1) full resolution is reached at which point each strain will have a unique haplotype and the set resolution score is zero; (2) when none of the remaining patterns are able to improve the current resolution of the set; (3) when some predefined number of sites or resolution threshold is reached; (4) no more patterns remain.

4 $$ r = \sum\limits_{i=0}^{\max(\mathbf{p})} s_{i}^{2} - s_{i} ; \\  $$

where **p** is a pattern vector and *s*_*i*_ is the number of strains in the *i*^th^ feature category.

$$\mathbf{p}_{\text{update}} = \left[f(p_{t1} \times p_{s1}), f(p_{t2} \times p_{s2}), \dotsc, f(p_{tn} \times p_{sn})\right] ; $$

where *p*_*ti*_×*p*_*si*_ is the cartesian product between sets in a pattern vector, *p*_*t*_, and the current minimum spanning set pattern vector, *p*_*s*_.

5 $$ {\selectfont{\begin{aligned} a &= \left\{p_{ti} \times p_{si} \forall i \in \{1,2, \dotsc, n\} \colon \vert p_{ti} \times p_{si} \vert = 1\right\} \\[1em] {}f\left(p_{t} \times p_{s}; a\right) &= \left\{\begin{array}{ll} g(p_{t} \times p_{s}; a) \quad & \text{if}\ g(p_{t} \!\times\! p_{s}; a) \neq \emptyset \\[1.5em] f(p_{t} \times p_{s}; a\cup (p_{t}\times p_{s})) \quad & \text{otherwise} \end{array}\right. \\ \end{aligned}}}  $$

Assuming there are no missing or ambiguous calls, Eq. 5 simplifies to:

6 $$ {}f_{s}(p_{t} \times p_{s}; a\! = \!\{p_{ti} \times p_{si} \forall i \in \{1, 2, \dotsc, n\}\}) \!\mapsto\! \{i \colon a_{i} \!\in \!(p_{t} \times p_{s}) \}   $$

If multiple patterns tie for the best score, the one that is further up in the original sorted list is chosen because it will provide the greatest redundancy in the final set of patterns. This is due to the fact that higher ranking patterns offer more diversity, and therefore are more likely to complement other patterns in the set and partially compensate for them if they are missing. This added tolerance is beneficial because some of the targets might not be successfully amplified and sequenced.

As patterns are added, their associated amplicons are checked for overlap with the amplicons that are already included in the set. If a conflict cannot be resolved by removing one of the amplicons, then the new pattern is skipped and the pattern with the next best score is added and checked.

### Customizing the VaST workflow

Several user-defined parameters change the way the Pattern Selection Module handles the input data. Certain strains that are included in the preprocessing step can be marked for removal and will therefore not be considered in determining the final resolution. Lists of variant sites can be flagged either for removal or for mandatory inclusion in the final set. By default, VaST attempts to achieve maximum strain resolution; however, there are settings which will force VaST to stop once a certain number of amplicons have been added or when a resolution threshold has been met. Finally, an additional input array may be supplied which defines an alternative resolution objective. By default, VaST will not prioritize the resolution of any particular strains. If an alternative resolution objective is provided, VaST will favor patterns that help attain the alternative resolution before attempting full resolution. Alternative resolution objectives are useful when it is more critical to resolve certain strains over others. To summarize, VaST can be run using any of the following workflow options: the full workflow which provides full strain resolution using any of the amplicon candidates, the abridged workflow which stops once a user-specified number of amplicons are added or a resolution threshold is met, the weighted workflow which prioritizes the resolution of certain groups of strains using an alternative resolution objective, and the set extension workflow which appends to an existing set of targets.

## Results

### Benchmarking

**Fig. 2:**
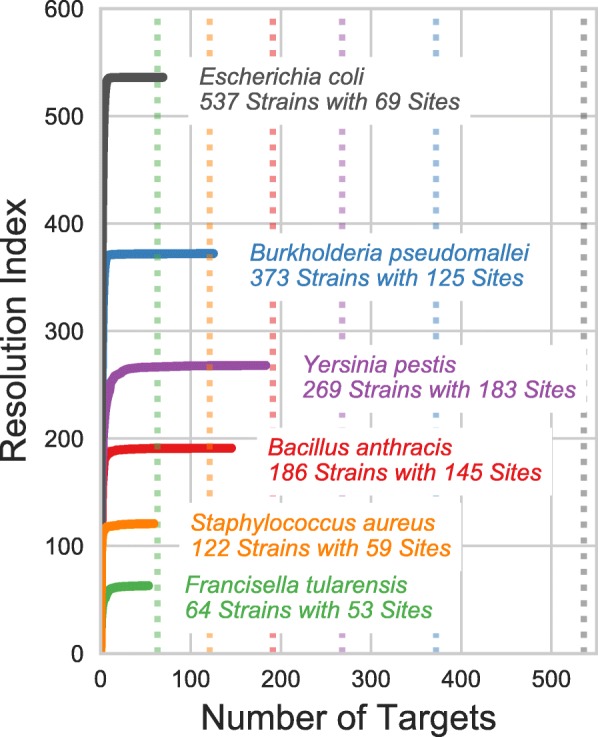
Most of the resolution is achieved within the first few targets. Minimum spanning sets were generated for strains of *Bacillus anthracis*, *Burkholderia pseudomallei*, *Escherichia coli*, *Francisella tularensis*, *Staphylococcus aureus*, and *Yersinia pestis*. The plot shows how the resolution index (*N*_strains_− average group size±*SD*) increases with each additional site.The number of differentiable strains included in the panel design and the size of the minimum spanning set is indicated next to each plot. The dashed vertical lines indicate the number of sites expected in the worst-case (*N*−1 sites)

We benchmarked VaST’s performance using 6 bacterial strain complexes: 537 strains of *Escherichia coli* using 189,570 SNPs, 373 strains of *Burkholderia pseudomallei* using 94,647 SNPs, 269 strains of *Yersinia pestis* using 11,249 SNPs, 186 strains of *Bacillus anthracis* using 11,989 SNPs, 64 strains of *Francisella tularensis* using 16,720 SNPs, and 122 strains of *Staphylococcus aureus* using 169,382 SNPs. These pathogens were chosen based on their relevance to human disease outbreaks and their potential for use as biothreat agents. The strains we used were drawn from previously published and well-established strain complexes [44–47]. We generated minimum spanning sets for each strain complex to demonstrate how well VaST performs in a number of genomic contexts. The *E. coli* minimum spanning set was the most efficient by resolving all 537 strains with only 69 amplicons which is 88% fewer than the number required in the worst case (dotted gray line in Fig. 2). For the other species, the number of required sites was relatively higher, providing only a 66%, 52%, 32%, 22%, and 17% reduction in the number of required sites over the worst case for *B. pseudomallei*, *Staphylococcus aureus*, *Y. pestis*, *B. anthracis*, and *F. tularensis*, respectively. The resolution index — the difference between the number of strains and the average unresolved group size — increases dramatically within the first few sites which suggests that most of the resolution is achieved early on, generally within the first 20 sites for the species we tested. The remaining sites typically resolve only a couple of strains each.

**Fig. 3:**
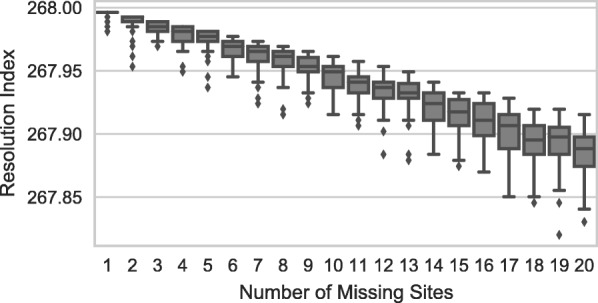
The redundancy built into the minimum spanning set design makes it tolerant to missing sites. The plot shows how well the *Yersinia pestis* minimum spanning set tolerates missing sites. The x-axis is the number of missing sites and the y-axis is the expected resolution index. Each box-plot shows the distribution of resolution values for different panels (*N*=50) with 1 to 20 sites randomly removed. The resolution index of the full panel is 269 and the median resolution when 20 sites are missing is 267.9 — a difference of only 1.1

The haplotype-based approach to building a minimum spanning set (as opposed to using a single unique marker to identify each strain) adds a large amount of redundancy. For example, no matter how early in the set a strain is resolved, its haplotype will still consist of all the target sites (e.g. Fig. 1j, strain 4). Similarly, if two strains are not resolved until the last site, all of the previous sites are redundant and do not provide any useful information for resolving the two strains (e.g. Fig. 1j, strains 1 & 2). All of this redundancy is useful because it makes the set more robust to missing targets. This is evident in Fig. 3 which shows how tolerant the *Y. pestis* minimum spanning set is to an increasing number of missing sites. Even when different combinations of 20 sites are missing, the median resolution index is 267.9 which is only slightly lower than the maximum resolution index of 269.

The entire VaST pipeline can be run on a laptop computer. The preprocessing modules (Amplicon Filter and Pattern Discovery) require the most computing resources, but the amount of time and memory required is highly dependent on the size of the initial variant site matrix and whether or not strict mode is activated. As an example, using a single core of a laptop with a 2.4 GHz Intel Core i5 processor and 8GB of RAM, the preprocessing for the *Y. pestis* data set took approximately 4 hours. If more computing resources are available, VaST can use multiprocessing to speed up the preprocessing steps. The Pattern Selection module runs relatively quickly, and took under an hour for the *Y. pestis* data.

### Computational validation

We tested the performance of the full *Y. pestis* minimum spanning set using publicly available HTS data from NCBI’s Sequence Read Archive. We aligned reads generated from five different strains (Harbin35 (SRR1283952) [48], Pestoides B (SRR2177700) [49], Angola (SRR2153449) [50], Antiqua (SRR2176134) [51] from [52], and KIM10 (SRR2084698) [53] from [54]) to a reference genome (NC_003143.1 [55]) using bowtie2 [56] and analyzed the calls at each of the target locations. In all five cases the haplotype collected from the sequencing data matched the expected strain.

Sometimes samples will contain strains that were not a part of the original target panel design. To see how well the panel can perform when identifying such samples, we redesigned the *Y. pestis* panel after removing 5 of the original strains. The new panel required 176 sites to achieve full resolution and the removed strains were treated as if they were samples of new strains. Using the calls at the 176 target sites, we identified the strains that were most closely related to the sample strains based on how many of the calls matched. In each case, the strain that was the best match was also very closely related in the phylogentic tree (based on patristic distance) and the size of the clade that included both strains was small (Table 3).

**Table 3:** New strains that were not used to build the minimum spanning set are identified as closely related strains

Assembly accession	Strain name	Patristic distance	In same clade	Clade size
GCA_000255875.1 [61]	Biovar Orientalis AS200901434	2	Yes	3
GCA_000186725.1 [62]	Biovar Medievalis Harbin 35	7	Yes	2
GCA_000182545.1 [63]	Pestoides A	1	Yes	3
GCA_000006645.1 [64]	KIM10	9	Yes	2
GCA_000013805.1 [65]	Nepal516	10	Yes	3

The *Y. pestis* minimum spanning set was regenerated with 5 of the original strains removed. These strains were then treated as samples and identified using the new minimum spanning set. In each case, the strain that most closely matched the sample strain’s haplotype was closely related. The table shows the assembly accession and name of each of the strains that were removed. The patristic distance between the sample strain and the strain it was identified as was calculated using the full tree. The clade size is the size of the clade that included both strains

### Comparison to other methods

We compared the resolution achieved using VaST to the Loci Selector module of Panseq [35] to demonstrate how our approach is different. Using a matrix of 96 SNPs identified from *E. coli* O157:H7 [57], the LS module identified a collection of 20 SNPs that each individually offered the best discrimination for unique sets of strains. Combined, these 20 SNPs completely resolved 12 of the 19 strains, leaving a group of 7 unresolved strains. However, only 7 of the identified sites increased the resolution and the remaining 13 provided only redundant information. Because VaST prioritizes targets that evenly split up groups of strains rather than finding the most discriminatory targets at each step, it was able to completely resolve 13 strains (with a group of 6 remaining) using 6 sites. As the number of strains considered increases, we would expect an even larger improvement in performance.

**Fig. 4:**
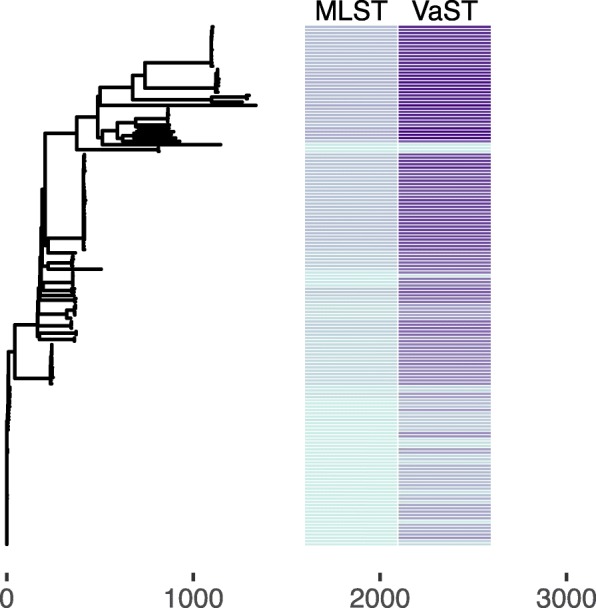
VaST identifies more targets than a traditional MLST and provides greater strain resolution. The neighbor joining tree was built using 5,000 SNPs from 159 strains of *Staphylococcus aureus*. The colors in the heatmap represent different strain groups ranging from 1-138. The MLST loci only resolved 41 groups as indicated by the smaller range of colors compared to VaST which resolved 138 groups

We also compared the strain resolution achieved with VaST to that of a traditional MLST assay using a total of 159 *S. aureus* whole genome sequences from the NCBI RefSeq database. Using these sequences, we generated a SNP matrix using NASP [41] and identified the ST from 7 housekeeping genes (*arcC, aroE, glpF, gmk, pta, tpi,* and *yqiL*) using an open-source MLST program (https://github.com/tseemann/mlst). A total of 41 different groups were resolved using MLST genes, with group sizes ranging from a single strain (*n*=20) to 44 strains and a mean size of 4.0. Using a total of 59 amplicons, VaST resolved 138 groups, with group sizes ranging from a single strain (*n*=122) to 8 strains and a mean size of 1.2. Figure 4 compares the resolution and it is clear that the VaST targets can resolve strains within very closely related groups.

### Experimental validation

We experimentally validated the *Y. pestis* minimum spanning set that VaST produced by performing a TAS assay. Due to the challenges associated with optimizing a multiplex PCR reaction for a large number of targets, we opted to use a truncated version of the panel which included only the first 42 amplicons. This truncated panel had a slightly lower resolution index (266.1 compared to 269 for the full panel) but it was able to resolve most of the major clades. Table 4 shows the number of unresolved groups of different sizes which were used to calculate the resolution index for the truncated panel. Using only 42 of the 183 sites, 38 strains can be uniquely identified (group size 1). The largest unresolved group consisted of 20 very similar biovar Orientalis strains that were all isolated from rodents in Peru. The median group size is 5 so at least half of the strains are in groups of 5 or smaller.

**Table 4:** Resolution of truncated Yersenia pestis minimum spanning set

Group size	Count
1	38
2	18
3	9
4	6
5	5
6	2
7	6
8	1
11	1
12	1
15	1
20	1

The table shows the expected resolution using only the first 42 of the 183-site *Y. pestis* minimum spanning set. The group size indicates a number of strains that could not be differentiated from one another and the count is how many groups of each size exist. A total of 28 strains were fully resolved and the largest group contained 20 unresolved strains

**Fig. 5:**
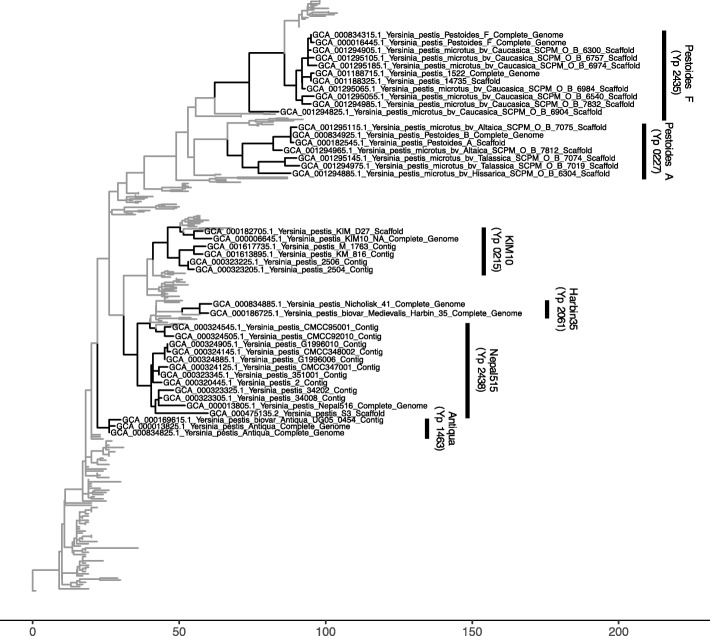
The *Y. pestis* samples were correctly identified using the target sites identified by VaST. The placement and resolution of the sample strains on a neighbor joining tree produced using the full SNP matrix (11,249 SNPs). The group of strains indicated for each sample represent the strains that were most similar to the sample strain at each of the targets analyzed in the truncated panel. The branch lengths indicate the number of SNP differences

The targets of the truncated minimum spanning set were amplified in sample DNA from six different *Y. pestis* strains (Pestoides A, Pestoides F, KIM10, Harbin35, Nepal515, and Antiqua) and the amplicons were sequenced. The calls made at each of the target sites placed every sample strain within the correct clade (Fig. 5). In each case, the maximum resolution expected for the minimum spanning set was achieved.

## Discussion

We have developed, benchmarked, and tested a desktop-compatible pipeline which identifies a minimum set of targets that are appropriate for bacterial strain identification. We anticipate that this software will aid in the design of customized, high-resolution typing assays that will be useful for forensic and epidemiological applications, or even for identifying and maintaining laboratory stocks of bacterial isolates. The minimum spanning algorithm implemented in VaST optimizes a combinatorially complex problem in a minimal amount of time even on a desktop computer. The haplotypes produced by VaST provide built-in redundancy which allows the panel to tolerate the likely failure of some amplicons without sacrificing much resolution. The many different run-time options available in VaST provide flexibility to accommodate many different situations. When some strains have particularly low coverage (lots of missing or ambiguous sites), turning off strict mode will open up many more target options for better results. On the other hand, when there is fairly even coverage across the strains, enabling strict mode will speed up the preprocessing steps. The set extension workflow can easily extend existing panels when additional strains or clades are identified or sequenced.

Compared to other strain typing methods, VaST offers a several advantages. Unlike the Pan-PCR method [34], VaST is able to take advantage of variation that exists in both coding and non-coding regions of the genome which provides a larger pool of options for strain differentiation. This is critical when expanding this approach to viral organisms. As a sequencing based approach, opposed to presence/absence detection, VaST is also able to maximize the information content of polymorphic sites, which makes it possible to detect the presence of previously unseen strains and place them within existing phylogenies. A failed target amplification in the Pan-PCR assay can easily corrupt the expected presence/absence signal and lead to a complete mis-characterization of a strain sample. In a VaST panel, the failure of certain targets will reduce resolution but will not result in a mis-identified strain.

The LS Module of PanSeq focuses on finding the variant sites that offer the most discriminatory power and thus it does not prioritize the addition of variants that are deeper in the phylogeny, as they resolve clades rather than individual strains. The resulting set of targets will therefore be less robust when new strains are introduced that were not a part of the panel design process. In contrast, VaST prioritizes sites that evenly split strain complexes at each step so that the early additions to the minimum spanning set tend to be more phylogenetically basal — stable variation that occurred earlier in the evolution of the organism. In essence, this approach seeks to resolve the full phylogeny, rather than just the leafs of the species tree. As a result, an important feature of VaST is its ability to characterize previously unseen strains, due to abundance of “deep” phylogenetic variants. This was demonstrated in our computation simulations which consistently place strains that were not included in the design of the panel into the correct clade with their most closely related neighbors.

Finally, over the last 20 years, a number of well validated variant markers and MLST profiles have been proposed for the purpose of identifying bacterial clades, particularly for identifying strains that are important in the biodefense sector and clinically relevant strains [30, 45, 58–60]. Using the information from previously established markers, VaST can add targets that are specifically designed to improve resolution, in a user-defined way, starting from the resolution provided by these markers. This allows for backwards compatibility and consistency with previous work thus avoiding the need to repeat the validation of well-established markers.

## Conclusions

Fine-scale resolution of bacterial strains is vital when narrowing down potential sources of a pathogen in forensic investigations, providing an accurate prognosis when diagnosing an infection, and establishing the transmission pattern of an infectious strain outbreak. As more and more strains are being identified and sequenced, it is important to be able to rapidly design, implement, and update strain identification panels. Strain typing using TAS technology can provide high resolution (hundreds or thousands of targets can be run simultaneously), scalability (many samples can be processed in a single sequencing run), and sensitivity (PCR amplification allows samples to be identified using small amounts of DNA). Using the ever-growing collection of variant sites identified through whole genome sequencing, VaST provides a tool which will automate the task of finding efficient strain typing markers for use in TAS panels.

## Availability and requirements

**Project Name:** VaST

**Project Home Page:** https://github.com/FofanovLab/VaST.git

**Operating system(s):** Platform independent

**Programming language:** Python

**Other requirements:** Anaconda (to use virtual environment)

**License:** MIT License

## Abbreviations

HTS: High-Throughput Sequencing
Indel: Insertion or deletion
PCR: Polymerase Chain Reaction
SNP: Single Nucleotide Polymorphism
TAS: Targeted PCR amplicon sequencing
VNTR: Variable Number Tandem Repeat
